# Depression as a Risk Factor for Dementia and Cognitive Decline in Type 2 Diabetes Mellitus: A Systematic Review of Longitudinal Studies

**DOI:** 10.7759/cureus.104825

**Published:** 2026-03-07

**Authors:** Dharmendra Kumar Gupta, Abu Md Mustaque, Rakesh Kumar

**Affiliations:** 1 Physiology, Burdwan Medical College, Burdwan, IND; 2 Psychiatry, Shri Ramkrishna Institute of Medical Sciences and Sanaka Hospital, Durgapur, IND; 3 Community Medicine, IQ City Medical College and Hospital, Durgapur, IND

**Keywords:** cognitive decline, dementia, depression, mild cognitive impairment, type 2 diabetes mellitus

## Abstract

Depression is highly prevalent among individuals with type 2 diabetes mellitus (T2DM) and has been increasingly recognized as a potential contributor to adverse cognitive outcomes. While both depression and diabetes independently increase the risk of cognitive decline and dementia, their combined impact on cognitive health remains an area of growing clinical interest. This systematic review aimed to evaluate the association between depression and cognitive outcomes in individuals with T2DM.

A systematic literature search was conducted in PubMed to identify relevant studies published between January 1, 2015, and December 31, 2025. The final search was performed on February 15, 2026. The search strategy combined Medical Subject Headings (MeSH) and free-text terms related to type 2 diabetes, depression, dementia, mild cognitive impairment, and cognitive decline. Studies were eligible if depression was assessed at baseline and cognitive outcomes were evaluated longitudinally through incident dementia diagnosis or repeated cognitive testing, enabling assessment of cognitive decline trajectories.

Six studies met the predefined eligibility criteria and were included in the qualitative synthesis. Across the included studies, depression was consistently associated with adverse cognitive outcomes in individuals with T2DM. Longitudinal cohort studies demonstrated increased risk of incident dementia, with reported hazard ratios ranging approximately from 2.17 to 2.59. Other studies reported accelerated cognitive decline or increased risk of mild cognitive impairment among individuals with coexisting depression and diabetes.

Depression appears to be an important and potentially modifiable risk factor for cognitive decline and dementia among individuals with type 2 diabetes. Early identification and effective management of depressive symptoms in patients with diabetes may help reduce the risk of adverse cognitive outcomes and support preservation of cognitive health in this high-risk population.

## Introduction and background

Type 2 diabetes mellitus (T2DM) and depression are among the most prevalent chronic disorders worldwide and frequently coexist in clinical settings. Individuals living with diabetes experience nearly double the risk of developing depressive symptoms compared with the general population, reflecting a complex bidirectional relationship mediated by behavioral, neuroendocrine, and inflammatory pathways [1,2]. Depression in patients with diabetes has been consistently associated with poor glycemic control, reduced treatment adherence, higher rates of microvascular and macrovascular complications, and increased mortality. Beyond psychological distress alone, comorbid depression contributes substantially to the overall disease burden and functional impairment in this population. Approximately one in four individuals with type 2 diabetes experience clinically significant depressive symptoms, highlighting the substantial burden of psychiatric comorbidity in this population [3].

In parallel, increasing attention has been directed toward the cognitive consequences of diabetes. A large body of epidemiological and mechanistic evidence suggests that T2DM accelerates brain aging and increases susceptibility to both vascular and neurodegenerative pathology. Chronic hyperglycemia, insulin resistance, endothelial dysfunction, oxidative stress, and low-grade systemic inflammation have all been implicated in neuronal injury and cerebral microvascular damage. Meta-analytic findings indicate that diabetes confers approximately a 1.5-2-fold higher risk of incident dementia, including both Alzheimer’s disease and vascular dementia, thereby establishing diabetes as an important modifiable risk factor for cognitive decline [4].

Depression itself has also emerged as an independent predictor of adverse cognitive outcomes. Longitudinal cohort studies demonstrate that depressive symptoms in midlife and late life are associated with faster decline in memory and executive function, increased risk of mild cognitive impairment, and higher incidence of dementia [5]. Proposed mechanisms include hypothalamic-pituitary-adrenal axis dysregulation, glucocorticoid neurotoxicity, reduced neurotrophic support, systemic inflammation, and cerebrovascular compromise. These biological processes substantially overlap with the metabolic and vascular abnormalities observed in diabetes, suggesting that the coexistence of both conditions may exert additive or even synergistic deleterious effects on brain health [6].

Given that depression and T2DM frequently co-occur, their combined influence on cognition is of particular clinical concern. Individuals affected by both disorders are exposed simultaneously to metabolic stress, vascular injury, and psychosocial adversity, potentially amplifying vulnerability to neurodegeneration. Population-based investigations have reported that the coexistence of diabetes and depression confers a greater risk of dementia than either condition alone, implying a compounded effect rather than independent contributions [7]. Despite these observations, much of the existing literature has examined depression or diabetes in isolation or has included heterogeneous populations without specifically focusing on individuals with established T2DM. Consequently, the incremental impact of depression within diabetic cohorts remains incompletely understood.

Over the past decade, a limited but methodologically rigorous set of longitudinal studies has begun to address this gap. Prospective cohorts of patients with T2DM have shown that baseline depressive symptoms are associated with increased incidence of dementia, greater likelihood of mild cognitive impairment and Alzheimer’s disease, and accelerated cognitive decline over time [8-11]. Additional evidence from neuropsychological and neuroimaging studies also suggests an association between depressive symptoms and poorer cognitive performance among individuals with diabetes [12]. Recent trajectory analyses further suggest that depression may influence transitions toward dementia states in this population [13]. However, differences in depression assessment tools, cognitive outcome measures, and confounder adjustment across studies have resulted in variability in reported effect estimates, and these findings have not yet been systematically synthesized.

Clarifying whether depression independently contributes to dementia risk in patients with T2DM has important implications for clinical practice and prevention strategies. If depression represents a modifiable risk factor for cognitive decline within diabetes, timely identification and management of depressive symptoms may offer an opportunity to mitigate long-term neurological outcomes. To date, no systematic review has specifically synthesized longitudinal evidence examining depression as a risk factor for cognitive outcomes exclusively within individuals with established type 2 diabetes. Clarifying this relationship may help identify depression as a potentially modifiable target for dementia prevention in this high-risk group.

## Review

Methodology

A systematic literature search was conducted in PubMed to identify studies examining the association between depression and cognitive outcomes in individuals with type 2 diabetes. The search covered the period from January 1, 2015, to December 31, 2025, and the final search was performed on February 15, 2026. The search strategy combined Medical Subject Headings (MeSH) and free-text keywords related to type 2 diabetes, depression, and cognitive outcomes, including dementia, mild cognitive impairment, and cognitive decline. In addition, manual screening of reference lists of eligible articles and relevant reviews was performed to identify any additional studies that may not have been captured through database searching. This approach has been used in prior systematic reviews where the research question targets a highly specific clinical population and outcome.

A total of 145 records were identified (143 via PubMed and 2 through manual reference screening). After title/abstract screening and full-text eligibility assessment, six longitudinal cohort studies met predefined Population-Exposure-Comparator-Outcome-Study design (PECOS) criteria. Inclusion criteria required confirmed T2DM at baseline, depression assessed prior to cognitive outcomes, and longitudinal evaluation of incident dementia, mild cognitive impairment (MCI), or cognitive decline. Cross-sectional studies, non-T2DM cohorts, and studies without depression exposure assessment were excluded. Due to methodological heterogeneity in exposure definitions and outcome measures, a narrative synthesis approach was prespecified. The review methodology and reporting were guided by the Preferred Reporting Items for Systematic Reviews and Meta-Analyses (PRISMA) 2020 statement to ensure transparency, completeness, and reproducibility [14]. The search strategy and documentation of information sources were developed in accordance with the PRISMA-S extension for literature searches [15]. The protocol and eligibility criteria were predefined prior to study selection.

The review protocol was developed prior to study screening and data extraction. Although the protocol was not prospectively registered in PROSPERO, the eligibility criteria, exposure definitions, outcomes, and synthesis plan were predefined and were not modified during the review process. The methodological principles consistent with international prospective review standards were followed [16].

Eligibility Criteria

Studies were selected according to predefined Population-Exposure-Comparator-Outcome-Study (PECOS) design criteria. The population of interest included adults (≥18 years) with a diagnosis of type 2 diabetes mellitus at baseline, recruited from community-based or clinical cohorts. Studies exclusively involving type 1 diabetes, gestational diabetes, or mixed populations without separate T2DM data were excluded.

The exposure of interest was depression assessed at baseline, defined either by a clinical diagnosis based on standardized diagnostic criteria (DSM/ICD), physician documentation, or validated depressive symptom scales such as the Patient Health Questionnaire, Geriatric Depression Scale, Beck Depression Inventory, Hospital Anxiety and Depression Scale, or comparable instruments.

The comparator group consisted of individuals with T2DM without depression or with lower depressive symptom burden. Eligible outcomes included incident all-cause dementia, Alzheimer’s disease, vascular dementia, mild cognitive impairment (MCI), or objectively measured longitudinal cognitive decline assessed using standardized neuropsychological tests (e.g., MMSE, MoCA, memory, or executive function scores).

Only longitudinal observational designs, including prospective or retrospective cohort studies and nested case-control analyses within cohorts, were included to ensure temporal directionality between depression and cognitive outcomes. Cross-sectional studies, case reports, editorials, reviews, and conference abstracts were excluded. To reflect contemporary evidence, publications from January 2015 to December 2025 were considered. Only peer-reviewed articles published in English were included.

Information Sources and Search Strategy

A comprehensive electronic search was conducted primarily in PubMed due to its broad biomedical coverage and indexing of major clinical journals, supplemented by manual reference screening of included articles and relevant reviews to minimize the risk of missed studies. The search strategy combined controlled vocabulary (MeSH terms) and free-text keywords related to type 2 diabetes, depression, and cognitive outcomes.

The complete PubMed search strategy was: (“Diabetes Mellitus, Type 2”[Mesh] OR “type 2 diabetes mellitus” OR T2DM) AND (“Depression”[Mesh] OR depression OR “depressive symptoms” OR “major depressive disorder”) AND (“Dementia”[Mesh] OR dementia OR “Alzheimer Disease”[Mesh] OR “vascular dementia” OR “mild cognitive impairment” OR “cognitive decline”) AND (“Cohort Studies”[Mesh] OR cohort OR longitudinal OR prospective OR incidence OR follow-up), with filters applied for humans, English language, and publication years 2015-2025. The literature search covered studies published between January 1, 2015, and December 31, 2025. The search was last updated on February 15, 2026, using the same date limits to ensure inclusion of recently indexed studies within this timeframe.

Boolean operators (“AND”, “OR”) were used to optimize sensitivity and specificity. The complete search strategy was iteratively refined through pilot searches. In addition, reference lists of eligible articles and relevant reviews were manually screened to identify additional studies. Screening and eligibility assessment were performed independently by two reviewers, and disagreements were resolved through discussion and consensus. All records were exported to a citation manager, and duplicates were removed prior to screening. The detailed electronic search strategy is presented in Table 5 (Appendix). 

Study Selection

Study selection was conducted in two stages. First, titles and abstracts were screened for relevance. Articles clearly not meeting eligibility criteria were excluded at this stage. Second, full-text versions of potentially eligible studies were assessed in detail against the predefined criteria.

**Figure 1:**
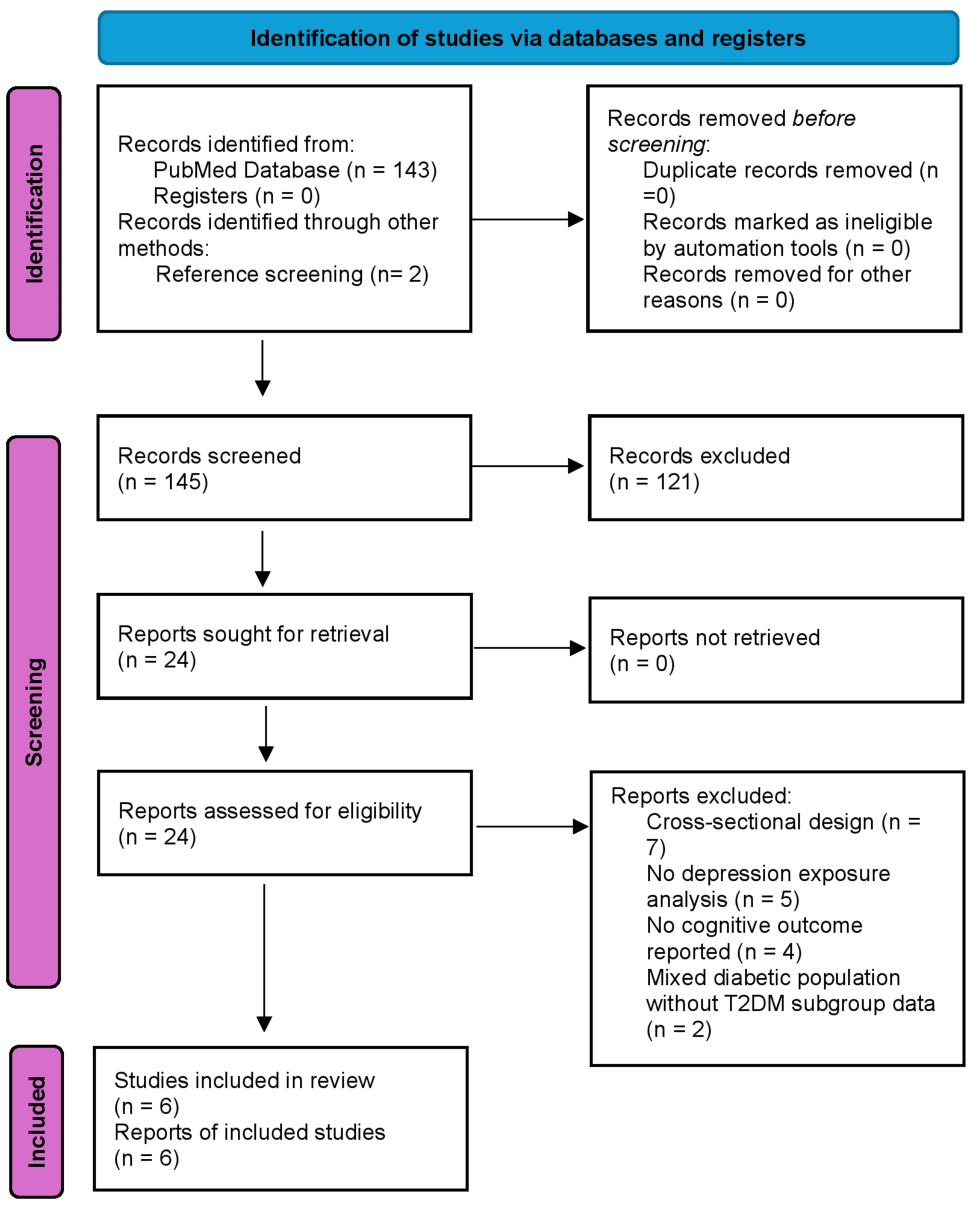
PRISMA flow diagram illustrating study identification, screening, eligibility assessment, and inclusion process.

Studies were included only when (i) T2DM status was confirmed at baseline, (ii) depression or depressive symptoms were assessed prior to outcome measurement, and (iii) cognitive outcomes were evaluated longitudinally. Discrepancies in eligibility assessment were resolved through discussion and consensus. The study selection process is illustrated in the PRISMA flow diagram (Figure 1).

Data Extraction

Data were extracted using a standardized form developed a priori. Extracted information included study characteristics (author, year, country, setting), sample size, participant demographics, diagnostic criteria for T2DM, method of depression assessment, cognitive outcome measures, duration of follow-up, covariates adjusted for in statistical models, and reported effect estimates (hazard ratios, relative risks, odds ratios, or regression coefficients with corresponding confidence intervals). When multiple statistical models were reported, the effect estimates from the most fully adjusted model were extracted to minimize confounding bias.

Data Management and Software

All records were exported to EndNote X9 (Clarivate Analytics, Philadelphia, PA, USA) for citation management and duplicate removal. Screening and data extraction were performed manually using Microsoft Excel 2021 (Microsoft Corporation, Redmond, WA, USA). The PRISMA flow diagram was constructed using Microsoft PowerPoint 2021. No statistical software was used, as quantitative meta-analysis was not performed.

Risk of Bias Assessment

Risk of bias was assessed using the Newcastle-Ottawa Scale (NOS), which remains widely used in systematic reviews of observational cohort studies [17]. In addition to the overall star ratings, domain-level considerations, including cohort selection, exposure ascertainment, outcome assessment, confounding adjustment, and follow-up completeness, were evaluated during the assessment process.

Each study was independently evaluated and assigned a score out of nine stars. Studies scoring 7-9 were considered low risk of bias, 5-6 moderate risk, and ≤4 high risk. Although alternative frameworks such as ROBINS-E provide a more detailed assessment of non-randomized exposure studies, the NOS was deemed appropriate given the small number of included studies and the need for consistent comparability [18]. Publication bias was not formally assessed due to the small number and heterogeneity of included studies.

Data Synthesis

Given the small number of eligible studies and heterogeneity in exposure definitions, cognitive outcomes, and effect measures, a narrative synthesis was planned a priori. Quantitative meta-analysis was not performed due to insufficient methodological homogeneity across studies.

Certainty of Evidence

The overall certainty of evidence for each outcome was evaluated using the Grading of Recommendations Assessment, Development and Evaluation (GRADE) framework [19]. Observational studies were initially considered low-certainty evidence and were subsequently rated up or down based on risk of bias, consistency of findings, directness, precision, and potential publication bias. Certainty levels were categorized as high, moderate, low, or very low. The certainty of evidence was rated as moderate, reflecting a consistent direction of association across multiple longitudinal cohort studies with large sample sizes, although the observational design of the included studies limits causal inference.

Results

Of the 145 identified records, 121 were excluded during title and abstract screening primarily due to cross-sectional design, absence of depression exposure assessment, or non-T2DM populations. Twenty-four full-text articles were assessed for eligibility. Eighteen studies were excluded for the following reasons: cross-sectional design (n=7), depression exposure not assessed at baseline (n=4), no eligible longitudinal cognitive outcome reported (n=3), mixed diabetic populations without separate T2DM subgroup analysis (n=2), duplicate cohort publication or overlapping dataset (n=1), and insufficient effect estimates for interpretation (n=1). The final synthesis included six longitudinal cohort studies. No eligible randomized or interventional studies were identified.

The included studies were conducted across diverse settings, including Denmark, Scotland, the United Kingdom, Italy, and the United States, with sample sizes ranging from 655 to over 2.4 million participants and follow-up periods between 4 and 10 years. Depression was assessed using clinical diagnoses or validated symptom scales, and cognitive outcomes included incident dementia, mild cognitive impairment, and longitudinal cognitive decline (Table 1) [7-11,13].

**Table 1:** Characteristics of included longitudinal studies evaluating depression and cognitive outcomes among adults with type 2 diabetes. CES-D: Center for Epidemiologic Studies Depression Scale; CESD-10: 10-item Center for Epidemiologic Studies Depression Scale; HADS-D: Hospital Anxiety and Depression Scale–Depression subscale; MCI: mild cognitive impairment; MRI: magnetic resonance imaging; ET2DS: Edinburgh Type 2 Diabetes Study; ELSA: English Longitudinal Study of Ageing; T2DM: type 2 diabetes mellitus

Study (Author, Year)	Country/Setting	Design	Sample size (n)	Age	Follow-up	Depression assessment	Cognitive outcome
Katon 2015 [7]	Denmark (national registry)	Population-based cohort	2,454,532	≥50 years	up to 6 years	Hospital/registry depression diagnosis	Incident dementia
Carr 2021 [8]	Scotland (ET2DS cohort)	Prospective cohort	1,066	60–75 years	median 10.6 years	HADS-D scale	Incident dementia
Johnson 2015 [9]	USA (HABLE/FRONTIER/TARCC)	Prospective cohort	2,436	older adults	~5 years	Cohort-defined depression measures	MCI and Alzheimer’s disease
Demakakos 2017 [10]	UK (ELSA)	Prospective cohort	10,524	≥50 years	8–10 years	CES-D scale	Memory & executive decline
Verhagen 2022 [11]	Multinational (CAROLINA-COGNITION)	Trial-based longitudinal cohort	3,163	64 years	up to 6 years	Depressive symptom scale	Global cognitive decline
Lenzi 2024 [13]	Italy	Administrative retrospective cohort	11,441	adults	up to 7 years	Major depression diagnosis	Dementia transition trajectories

An important methodological consideration across the included studies relates to the heterogeneity of depression and cognitive assessment instruments. Depression was identified either through clinical diagnostic criteria (DSM/ICD) or through validated symptom-based scales such as the Hospital Anxiety and Depression Scale (HADS), the Center for Epidemiologic Studies Depression Scale (CES-D and CESD-10), the Patient Health Questionnaire-9 (PHQ-9), the Geriatric Depression Scale (GDS), and the Beck Depression Inventory (BDI) [20-27]. These instruments have been widely validated in clinical and epidemiological settings and demonstrate acceptable psychometric properties for detecting depressive symptom burden. Cognitive outcomes were assessed using standardized tools such as the Mini-Mental State Examination (MMSE) and the Montreal Cognitive Assessment (MoCA), both of which are established screening measures for global cognition and mild cognitive impairment [23,24]. Although the use of validated instruments strengthens internal validity, variability in cut-off thresholds and scale sensitivity across studies may have contributed to heterogeneity in reported effect estimates.

Katon et al. demonstrated that individuals with comorbid depression and diabetes had a higher risk of developing dementia compared with individuals without either condition (HR: 2.17, 95% CI 2.10-2.24) [7]. Carr et al. reported that depressive symptoms were associated with a significantly increased risk of incident dementia among individuals with type 2 diabetes, with an adjusted hazard ratio (HR) of 2.59 (95% CI 1.62-4.15) over a median follow-up of approximately 10 years [8]. Johnson et al. found that the coexistence of depression and diabetes significantly increased the risk of mild cognitive impairment (OR: 3.01, 95% CI 1.36-6.67) [9]. Demakakos et al. reported accelerated memory decline among participants with both diabetes and elevated depressive symptoms (β: −0.27, 95% CI −0.45 to −0.08 per study wave) [10]. Verhagen et al. observed an increased risk of accelerated cognitive decline in individuals with type 2 diabetes and depressive symptoms (OR: 1.27, 95% CI 1.08-1.49) [11]. Lenzi et al., using a multi-state survival model, demonstrated that depression increased the probability of subsequent dementia during follow-up among individuals with type 2 diabetes [13].

Methods of depression ascertainment varied across studies. Some studies defined depression using clinician diagnosis or administrative/medical record indicators, whereas others used validated depressive symptom scales with established thresholds or continuous symptom burden. In all included studies, depression (or depressive symptom burden) was measured at baseline or prior to the longitudinal cognitive outcome assessment, consistent with the eligibility requirement of temporality. Definitions of depression exposure, comparator groups, cognitive outcomes, and reported effect measures varied across studies. Most cohorts defined depression using clinical diagnoses or validated symptom scales, while outcomes ranged from incident dementia to changes in neuropsychological performance. Detailed exposure definitions and effect metrics are summarized in Table 2.

**Table 2:** Summary of associations between depression and cognitive outcomes in individuals with type 2 diabetes in the included longitudinal studies. HR: hazard ratio; OR: odds ratio; CI: confidence interval; β: regression coefficient; HADS-D: Hospital Anxiety and Depression Scale–Depression subscale; CES-D: Center for Epidemiologic Studies Depression Scale; MCI: mild cognitive impairment

Study	Participants(N)	Depression Measure	Outcome	Effect Estimate
Katon 2015 [7]	2.45 million	Clinical diagnosis	Incident dementia	HR: 2.17 (2.10–2.24)
Carr 2021 [8]	1,066	HADS-D ≥8	Incident dementia	HR: 2.59 (1.62–4.15)
Johnson 2015 [9]	2,436	Clinical depression	MCI risk	OR: 3.01 (1.36–6.67)
Demakakos 2017 [10]	10,524	CES-D ≥4	Cognitive decline	Β: −0.27 (−0.45 to −0.08)
Verhagen 2022 [11]	3,163	CES-D symptoms	Accelerated cognitive decline	OR: 1.27 (1.08–1.49)
Lenzi 2024 [13]	11,441	Clinical diagnosis	Dementia transition	Depression increased dementia probability to 3.7%

Cognitive outcomes were assessed using two broad approaches. First, several studies examined incident clinical outcomes, including dementia and/or mild cognitive impairment, using diagnostic codes, clinical diagnosis, or adjudicated clinical assessment [7-9,13]. Second, other studies evaluated longitudinal cognitive performance using repeated neuropsychological measures to model cognitive trajectories and rates of decline [10,11].

Across the included longitudinal studies, the primary potential sources of bias related to heterogeneity in depression measurement (diagnostic indicators versus symptom scales), differences in outcome ascertainment (registry-based dementia diagnosis versus repeated cognitive testing), and variability in covariate adjustment models. Overall, the included studies were considered methodologically suitable for qualitative synthesis given the longitudinal design and clear temporality between exposure and outcomes.

All included studies reported an association between baseline depression or higher depressive symptom burden and adverse subsequent cognitive outcomes among adults with T2DM, either as an increased risk of incident dementia/MCI or as faster cognitive decline over follow-up. Studies evaluating incident dementia/MCI consistently reported higher event risk among participants with depression compared with those without depression [7-9,13]. Studies assessing cognition longitudinally reported steeper decline or poorer trajectories among participants with depressive symptoms [10,11].

Methodological quality was generally high. Five studies were rated as low risk of bias, while one study demonstrated moderate risk due to limited comparability adjustment. Newcastle-Ottawa Scale scores ranged from 6 to 9, indicating an overall robust study design (Table 3).

**Table 3:** Risk of bias assessment of included longitudinal studies using the Newcastle–Ottawa Scale (NOS).

Study (Author, Year)	Selection (4)	Comparability (2)	Outcome (3)	Total (/9)	Risk category
Katon 2015 [7]	4	2	3	9	Low
Carr 2021 [8]	4	2	3	9	Low
Johnson 2015 [9]	3	2	2	7	Low
Demakakos 2017 [10]	3	2	2	7	Low
Verhagen 2022 [11]	3	2	2	7	Low
Lenzi 2024 [13]	4	2	3	9	Low

Using the Grading of Recommendations Assessment, Development and Evaluation (GRADE) framework, the overall certainty of evidence for the association between depression and adverse cognitive outcomes in T2DM was rated as moderate. Although all included studies were observational cohort designs, the certainty was upgraded due to consistent direction of effects across studies, large cumulative sample sizes, clear temporal sequencing between depression and cognitive outcomes, and biological plausibility. Minor heterogeneity in exposure definitions and outcome measures prevented a high-certainty rating. Detailed GRADE assessments for each outcome are presented in Table 4.

**Table 4:** Certainty of evidence assessment using the GRADE framework for the association between depression and adverse cognitive outcomes in adults with type 2 diabetes mellitus. GRADE: Grading of Recommendations Assessment, Development and Evaluation; MCI: mild cognitive impairment; T2DM: type 2 diabetes mellitus

Outcome	No. of studies	Study design	Risk of bias	Inconsistency	Indirectness	Imprecision	Overall certainty (GRADE)	Interpretation
Incident dementia / MCI	4	Longitudinal cohort	Not serious	Not serious	Not serious	Not serious	Moderate	Depression likely increases risk of dementia/MCI in T2DM
Cognitive decline trajectories	2	Longitudinal cohort with repeated testing	Not serious	Minor heterogeneity	Not serious	Not serious	Moderate	Depression likely associated with faster cognitive decline
Overall cognitive outcomes (combined narrative synthesis)	6	Longitudinal cohort	Not serious	Minor heterogeneity	Not serious	Not serious	Moderate	Consistent evidence supporting depression as a risk factor

Discussion

In this systematic review, we synthesized longitudinal evidence examining the association between comorbid depression and subsequent cognitive outcomes among adults with type 2 diabetes mellitus (T2DM). Across six longitudinal cohort studies, baseline depression or elevated depressive symptom burden was consistently associated with an increased risk of incident dementia, mild cognitive impairment, or accelerated cognitive decline. Importantly, this association was observed despite substantial heterogeneity in study populations, depression assessment methods, and cognitive outcome measures, underscoring the robustness of the observed relationship.

Studies evaluating incident dementia outcomes demonstrated that individuals with T2DM and comorbid depression had a significantly higher risk of developing dementia compared with their non-depressed counterparts [7-9,13]. These associations persisted after adjustment for key demographic, metabolic, and vascular confounders, suggesting that depression appears to be independently associated with increased dementia risk within diabetic populations. Complementing these findings, longitudinal investigations focusing on cognitive trajectories consistently reported faster decline in global cognition, memory, or executive function among participants with depressive symptoms [10,11].

Together, these findings indicate that depression is not merely a correlate of cognitive impairment in T2DM but appears to be independently associated with subsequent cognitive deterioration. The consistency in effect direction across diverse cohorts strengthens confidence in the validity of this association.

The present findings align with broader epidemiological evidence demonstrating that both T2DM and depression independently increase the risk of dementia [4,5]. However, prior studies have largely examined these conditions in isolation or within heterogeneous populations. By restricting inclusion to cohorts with established T2DM and longitudinal assessment of cognitive outcomes, the current review extends previous work by clarifying the incremental contribution of depression within diabetic populations.

Our results are also concordant with population-based studies showing additive or synergistic effects of diabetes and depression on dementia risk [7]. The observation that depression remains a significant predictor even after accounting for glycemic control, vascular comorbidity, and socioeconomic factors suggests that its impact is not solely mediated through traditional cardiometabolic pathways.

Several overlapping mechanisms may plausibly explain the observed association between depression and adverse cognitive outcomes in T2DM. Depression is characterized by dysregulation of the hypothalamic-pituitary-adrenal axis, resulting in chronic glucocorticoid exposure, which has been linked to hippocampal atrophy and impaired neurogenesis.

Additionally, both depression and diabetes are associated with systemic low-grade inflammation, endothelial dysfunction, and insulin resistance, processes that contribute to cerebrovascular injury and neurodegeneration [6].

Behavioral and psychosocial factors may further exacerbate risk. Depression in T2DM is associated with poorer self-care behaviors, reduced physical activity, suboptimal medication adherence, and social withdrawal, all of which may indirectly accelerate cognitive decline. Neuroimaging evidence demonstrating structural brain changes in depressed individuals with diabetes lends further support to the biological plausibility of a direct brain effect [12].

The findings of this review have important implications for clinical practice. Depression is common, underdiagnosed, and potentially treatable in patients with T2DM. The consistent association between depression and adverse cognitive outcomes suggests that routine screening for depressive symptoms in diabetes care may identify individuals at elevated risk for cognitive decline. Early recognition and management of depression could therefore represent a pragmatic strategy to mitigate long-term neurological complications in this vulnerable population.

From a public health perspective, integrating mental health assessment into chronic disease management frameworks may help address modifiable risk factors for dementia, particularly in aging populations with a high burden of metabolic disease. Future studies incorporating standardized depression instruments, repeated cognitive assessments, and time-varying metabolic markers may help clarify temporal and causal pathways.

**Figure 2:**
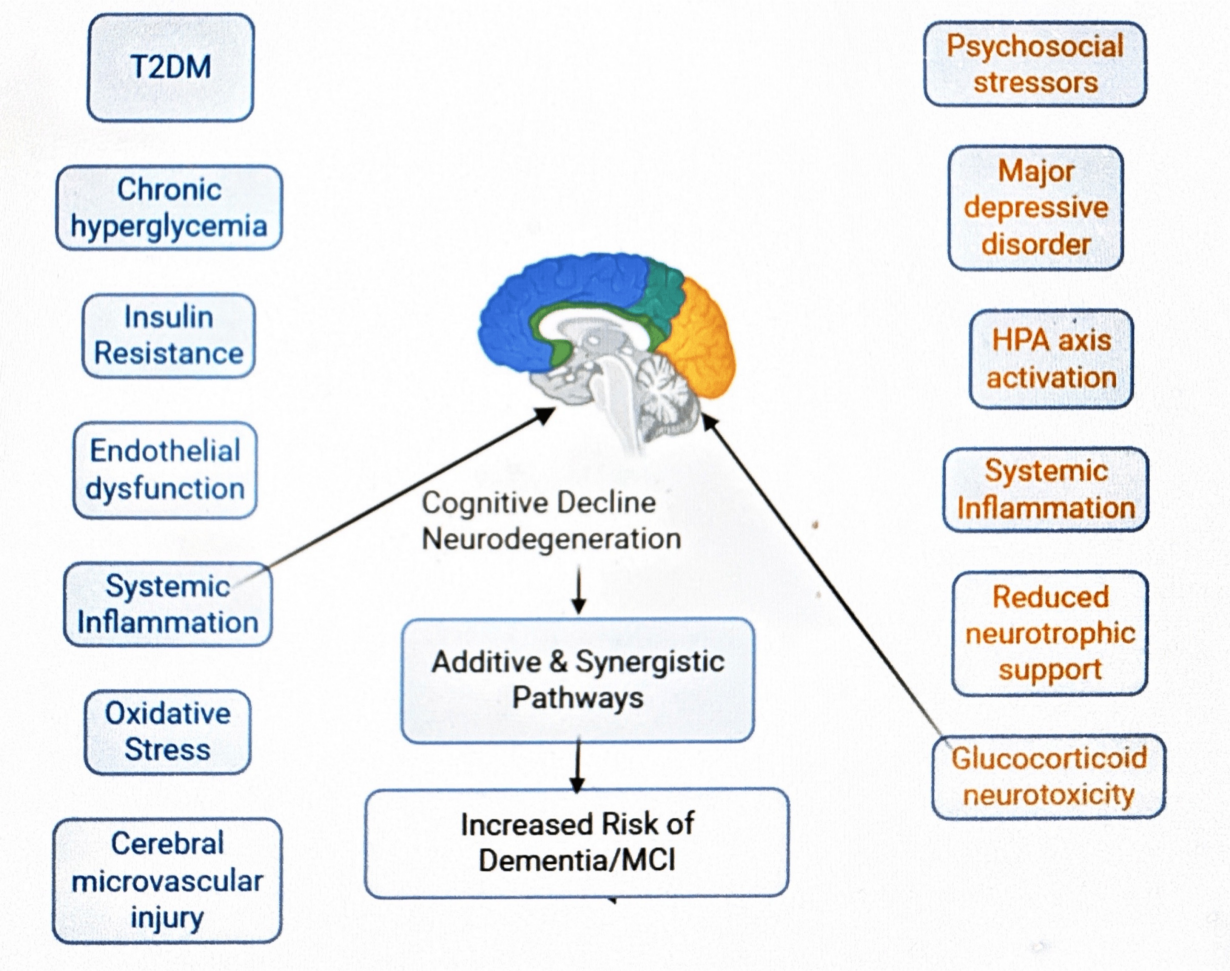
Proposed overlapping biological mechanisms linking type 2 diabetes mellitus and depression to accelerated cognitive decline. Metabolic and vascular abnormalities associated with T2DM and neuroendocrine and inflammatory pathways related to depression converge on the brain to promote neurodegeneration and increase the risk of dementia and mild cognitive impairment. The image is created using BioRender. T2DM: type 2 diabetes mellitus; HPA: hypothalamic–pituitary–adrenal; MCI: mild cognitive impairment

Type 2 diabetes mellitus (T2DM) contributes to chronic hyperglycemia, insulin resistance, endothelial dysfunction, oxidative stress, and systemic inflammation, leading to cerebral microvascular injury and neuronal damage. Depression is associated with hypothalamic-pituitary-adrenal (HPA) axis dysregulation, elevated glucocorticoid exposure, reduced neurotrophic support, and inflammatory activation. These additive and synergistic pathways converge to promote hippocampal atrophy, neurodegeneration, and increased risk of mild cognitive impairment (MCI) and dementia. The overlapping biological pathways linking T2DM and depression to accelerated neurodegeneration and cognitive decline are summarized schematically in Figure 2.

India currently represents one of the largest global populations of individuals living with T2DM, characterized by an earlier age of onset, higher insulin resistance, and greater cardiometabolic vulnerability compared with many Western populations. At the same time, depression remains substantially underdiagnosed and undertreated, particularly in patients presenting with chronic medical conditions. Cultural factors, stigma, and the tendency toward somatic presentation of depressive symptoms may further contribute to under-recognition in routine diabetes care settings [28,29].

South Asian populations, including Indians, demonstrate a distinctive metabolic phenotype characterized by a high prevalence of type 2 diabetes at younger ages and lower BMI thresholds than Western populations, accompanied by greater abdominal fat and lower muscle mass, which contribute to heightened cardiometabolic risk and earlier β-cell dysfunction [30]. Recent reviews suggest that reduced β-cell compensatory reserve and ectopic fat accumulation in the liver and muscle play an important role in this increased susceptibility. Compared with Caucasian and other ethnic groups, South Asians also show higher insulin resistance and a clustering of cardiometabolic risk factors that may amplify the impact of co-occurring depression on cognitive outcomes [31].

Given emerging evidence that comorbid depression may accelerate cognitive decline in individuals with T2DM, the implications for aging Indian populations are considerable. Dementia prevalence in India is projected to rise sharply in the coming decades, while structured cognitive screening within diabetes clinics remains uncommon. Integrating routine mental health assessment into diabetes management programs could therefore serve as a feasible and cost-effective strategy to identify individuals at elevated risk for future cognitive impairment. Collaborative care models involving psychiatrists, endocrinologists, and primary care physicians may be particularly beneficial in urban and semi-urban settings. Future longitudinal studies conducted in Indian cohorts are needed to determine whether the magnitude of depression-associated cognitive risk differs in South Asian populations and to explore potential sociocultural moderators of this relationship.

Clinical implications

The findings of this review have important clinical implications. Depression is highly prevalent among individuals with type 2 diabetes and may represent a potentially modifiable risk factor for cognitive decline and dementia. Early identification and management of depressive symptoms in patients with diabetes may therefore play a role in preserving cognitive health. Integrating routine depression screening into diabetes care could help identify individuals at higher risk for adverse cognitive outcomes and support timely multidisciplinary interventions.

Limitations

This review has several limitations. First, the literature search was conducted primarily in PubMed; although this database offers comprehensive biomedical coverage, relevant studies indexed exclusively in other databases may have been missed. Second, the number of eligible longitudinal studies was relatively small, reflecting limited research specifically examining depression-related cognitive risk within T2DM cohorts. Third, heterogeneity in depression assessment tools and cognitive outcome measures precluded quantitative meta-analysis. Fourth, registry-based studies may be subject to minor misclassification bias. Finally, most included studies were conducted in high-income countries, potentially limiting generalizability to low- and middle-income settings.

Future directions

Future research should prioritize large-scale prospective studies incorporating standardized depression diagnostic criteria, repeated cognitive assessments, and time-varying metabolic markers. Interventional studies examining whether effective treatment of depression attenuates cognitive decline in T2DM are particularly needed. Biomarker-based investigations exploring inflammatory mediators, hypothalamic-pituitary-adrenal axis activity, and neuroimaging correlates may help clarify mechanistic pathways. Longitudinal cohort studies conducted in South Asian and other low- and middle-income populations are essential to determine whether depression-associated cognitive risk differs across ethnic and sociocultural contexts.

## Conclusions

This systematic review provides consistent longitudinal evidence that comorbid depression in adults with type 2 diabetes mellitus is associated with an increased risk of incident dementia and accelerated cognitive decline. These findings highlight depression as a clinically meaningful and potentially modifiable risk factor for adverse cognitive outcomes in T2DM. Future large-scale prospective studies with standardized assessments of depression, cognition, and metabolic control are needed to clarify causal pathways and to determine whether effective treatment of depression can attenuate dementia risk in this high-risk population.

## Appendices

Detailed electronic search strategy

**Table 5:** Detailed electronic search strategy (supplementary Table 1).

Database	Search String	Filters Applied	Date Last Searched
PubMed	(“Diabetes Mellitus, Type 2” [Mesh] OR “type 2 diabetes mellitus” OR T2DM) AND (“Depression” [Mesh] OR depression OR “depressive symptoms” OR “major depressive disorder”) AND (“Dementia” [Mesh] OR dementia OR “Alzheimer Disease” [Mesh] OR “vascular dementia” OR “mild cognitive impairment” OR “cognitive decline”) AND (“Cohort Studies” [Mesh] OR cohort OR longitudinal OR prospective OR incidence OR follow-up)	Humans, English language, 2015–2025	15 February 2026

## References

[1] Sartorius N (2018). Depression and diabetes. Dialogues Clin Neurosci.

[2] Bădescu SV, Tătaru C, Kobylinska L (2016). The association between diabetes mellitus and depression. J Med Life.

[3] Wang F, Wang S, Zong QQ (2019). Prevalence of comorbid major depressive disorder in Type 2 diabetes: a meta-analysis of comparative and epidemiological studies. Diabet Med.

[4] Cao F, Yang F, Li J (2024). The relationship between diabetes and the dementia risk: a meta-analysis. Diabetol Metab Syndr.

[5] Byers AL, Yaffe K (2011). Depression and risk of developing dementia. Nat Rev Neurol.

[6] Hayley S, Hakim AM, Albert PR (2021). Depression, dementia and immune dysregulation. Brain.

[7] Katon W, Pedersen HS, Ribe AR (2015). Effect of depression and diabetes mellitus on the risk for dementia: a national population-based cohort study. JAMA Psychiatry.

[8] Carr AL, Sluiman AJ, Grecian SM (2021). Depression as a risk factor for dementia in older people with type 2 diabetes and the mediating effect of inflammation. Diabetologia.

[9] Johnson LA, Gamboa A, Vintimilla R (2015). Comorbid depression and diabetes as a risk for mild cognitive impairment and Alzheimer's disease in elderly Mexican Americans. J Alzheimers Dis.

[10] Demakakos P, Muniz-Terrera G, Nouwen A (2017). Type 2 diabetes, depressive symptoms and trajectories of cognitive decline in a national sample of community-dwellers: A prospective cohort study. PLoS One.

[11] Verhagen C, Janssen J, Biessels GJ (2022). Females with type 2 diabetes are at higher risk for accelerated cognitive decline than males: CAROLINA-COGNITION study. Nutr Metab Cardiovasc Dis.

[12] Raffield LM, Brenes GA, Cox AJ (2016). Associations between anxiety and depression symptoms and cognitive testing and neuroimaging in type 2 diabetes. J Diabetes Complications.

[13] Lenzi J, Messina R, Rosa S (2024). A multi-state analysis of disease trajectories and mental health transitions in patients with type 2 diabetes: a population-based retrospective cohort study utilizing health administrative data. Diabetes Res Clin Pract.

[14] Page MJ, McKenzie JE, Bossuyt PM (2021). The PRISMA 2020 statement: an updated guideline for reporting systematic reviews. BMJ.

[15] Rethlefsen ML, Kirtley S, Waffenschmidt S (2021). PRISMA-S: an extension to the PRISMA statement for reporting literature searches in systematic reviews. Syst Rev.

[16] Booth A, Clarke M, Dooley G (2012). The nuts and bolts of PROSPERO: an international prospective register of systematic reviews. Syst Rev.

[17] Stang A (2010). Critical evaluation of the Newcastle-Ottawa scale for the assessment of the quality of nonrandomized studies in meta-analyses. Eur J Epidemiol.

[18] Higgins JP, Morgan RL, Rooney AA (2024). A tool to assess risk of bias in non-randomized follow-up studies of exposure effects (ROBINS-E). Environ Int.

[19] Guyatt GH, Oxman AD, Vist GE (2008). GRADE: an emerging consensus on rating quality of evidence and strength of recommendations. BMJ.

[20] Zigmond AS, Snaith RP (1983). The hospital anxiety and depression scale. Acta Psychiatr Scand.

[21] Radloff LS (1977). The CES-D scale: a self-report depression scale for research in the general population. Appl Psychol Meas.

[22] Andresen EM, Malmgren JA, Carter WB, Patrick DL (1994). Screening for depression in well older adults: evaluation of a short form of the CES-D (Center for Epidemiologic Studies Depression Scale). Am J Prev Med.

[23] Folstein MF, Folstein SE, McHugh PR (1975). “Mini-mental state”: a practical method for grading the cognitive state of patients for the clinician. J Psychiatr Res.

[24] Nasreddine ZS, Phillips NA, Bédirian V (2005). The Montreal Cognitive Assessment, MoCA: a brief screening tool for mild cognitive impairment. J Am Geriatr Soc.

[25] Kroenke K, Spitzer RL, Williams JB (2001). The PHQ-9: validity of a brief depression severity measure. J Gen Intern Med.

[26] Yesavage JA, Brink TL, Rose TL (1983). Development and validation of a geriatric depression screening scale: a preliminary report. J Psychiatr Res.

[27] BE AT, WA CH, ME M (1961). An inventory for measuring depression. Arch Gen Psychiatry.

[28] Ali MK, Chwastiak L, Poongothai S (2020). Effect of a collaborative care model on depressive symptoms and glycated hemoglobin, blood pressure, and serum cholesterol among patients with depression and diabetes in India: The INDEPENDENT randomized clinical trial. JAMA.

[29] Khan F, Hussain S, Singh S (2025). A cross-sectional study on the prevalence and predictors of cognitive impairment and depression in elderly patients with type 2 diabetes mellitus. Cureus.

[30] Narayan KM, Kanaya AM (2020). Why are South Asians prone to type 2 diabetes? A hypothesis based on underexplored pathways. Diabetologia.

[31] Shah A, Kanaya AM (2014). Diabetes and associated complications in the South Asian population. Curr Cardiol Rep.

